# Facile Fluorescence Monitoring of Gut Microbial Metabolite Trimethylamine *N*-oxide via Molecular Recognition of Guanidinium-Modified Calixarene

**DOI:** 10.7150/thno.33459

**Published:** 2019-06-24

**Authors:** Huijuan Yu, Wen-Chao Geng, Zhe Zheng, Jie Gao, Dong-Sheng Guo, Yuefei Wang

**Affiliations:** 1 Institute of Traditional Chinese Medicine, Tianjin University of Traditional Chinese Medicine, Tianjin 301617, China; 2 College of Chemistry, Key Laboratory of Functional Polymer Materials (Ministry of Education), State Key Laboratory of Elemento-Organic Chemistry, Tianjin Key Laboratory of Biosensing and Molecular Recognition, Nankai University, Tianjin 300071, China

**Keywords:** calixarene, trimethylamine *N*-oxide, gut microbiota, fluorescence sensing, indicator displacement assay

## Abstract

Detection and quantification of trimethylamine *N*-oxide (TMAO), a metabolite from gut microbial, is important for the disease diagnosis such as atherosclerosis, thrombosis and colorectal cancer. In this study, a novel method was established for the sensing and quantitative detection of TMAO via molecular recognition of guanidinium-modified calixarene from complex matrix.

**Methods**: Various macrocycles were tested for their abilities to serve as an artificial TMAO receptor. Using the optimized receptor, we developed an indicator displacement assay (IDA) for the facile fluorescence detection of TMAO. The quantification of TMAO was accomplished by the established calibration line after excluding the interference from the various interfering substances in artificial urine.

**Results**: Among various macrocycles, water-soluble guanidinium-modified calix[5]arene (GC5A), which binds TMAO in submicromolar-level, was identified as the optimal artificial receptor for TMAO. With the aid of the GC5A•Fl (fluorescein) reporter pair, TMAO fluorescence “switch-on” sensing was achieved by IDA. The fluorescence intensity increased linearly with the elevated TMAO concentration. The detection was not significantly interfered by the various interfering substances. TMAO concentration in artificial urine was quantified using a calibration line with a detection limit of 28.88 ± 1.59 µM, within the biologically relevant low µM range. Furthermore, the GC5A•Fl reporter pair was successfully applied in analyzing human urine samples, by which a significant difference in fluorescence response was observed between the [normal + TMAO] and normal group.

**Conclusion:** The proposed supramolecular approach provides a facile, low-cost and sensitive method for TMAO detection, which shows promise for tracking TMAO excretion in urine and studying chronic disease progression in humans.

## Introduction

Trimethylamine *N*-oxide (TMAO) is the most focused metabolite originated from intestinal microorganisms, and its precursor, trimethylamine (TMA), is transformed from dietary carnitine, choline or choline-containing compounds by host intestinal bacteria 1, 2. TMA enters the liver via the portal circulation, where it is rapidly converted into a water-soluble compound, TMAO, by host hepatic flavin monooxygenases 1. In recent years, the mysterious veil of TMAO in the development of numerous diseases has been gradually uncovered 3-7. *In vivo* animal studies have shown that TMAO directly increased the reactivity and thrombotic potential of platelets, which are important risk factors for complications of cardiovascular metabolic diseases (e.g., heart attack and stroke) 3. In people, TMAO is mostly eliminated from body though urine, and is also excreted though breathe and sweat 8. As a noninvasive test method, urine testing is of great significance for the diagnosis and risk prediction of clinical diseases. Consequently, quantifying TMAO in urine becomes an indispensable and urgent clinical task that facilitates the early diagnosis of disease.

Some sophisticated methods have been reported for quantifying TMAO in biological matrices. With the high selectivity, sensitivity and throughput, mass spectrometry has been extensively applied in clinical detection. Undertaken to determine TMA reduced from TMAO by the guidance of complex protocols, gas chromatography-mass spectrometry (GC-MS) method suffered from the complicated and laborious procedures, time consumption, and incompleteness of TMAO transformation, limiting its clinical use 9. To our knowledge, many researchers preferred utilization of liquid chromatography tandem mass spectrometry (LC-MS/MS) for determining TMAO, employing a stable isotopically labelled standard 10-16, which required synthetic stable isotope markers, specially trained personnel, and specialized and costly analytical instruments that were not widely available in clinical diagnostic laboratories. Other studies have employed proton nuclear magnetic resonance spectroscopy (^1^H NMR) to determine TMAO, which underwent poor sensitivity, instability of determining results subjected to pH variation 17-19. To address these issues, the development of a novel, simple, inexpensive and rapid method for TMAO detection is urgently needed.

The well-developed artificial receptors with discrete cavities, macrocyclic hosts of cyclodextrin, calixarene and cucurbituril types, selectively bind certain guests. With the help of the intriguing host-guest properties between macrocycles and biological substrates, the molecular recognition by macrocycles in aqueous media has attracted a great deal of attention. It has been used in disease diagnosis and therapy 20-30, enhancement of drugs' solubility and stability 31-34, and regulation of protein-protein interactions 35, 36, among others.

Recently, researchers have increasingly paid attention to calixarene macrocycles in the biomedical field on account of their recognition and assembly properties 24-26, 37-41. To the best of our knowledge, despite the significant achievements in the molecular recognition of macrocycles, the binding and detection of TMAO by macrocycle have not been reported yet. In this study, we explored the facile fluorescence monitoring for TMAO on the basis of indicator displacement assay (IDA). Water-soluble guanidinium-modified calix[5]arene (GC5A) was screened as an artificial receptor from various water-soluble macrocycles, including cyclodextrins, *p*-sulfonatocalixarenes and cucurbiturils. Benefiting from submicromolar binding of TMAO by GC5A, the fluorescence “switch-on” sensing of TMAO was realized with high sensitivity. It was impressed us that the quantitative detection of TMAO was achieved in artificial urine within the biologically relevant low μM range without tedious sample pretreatment, conducing to point-of-care testing. Also, the fluorescence response of human urine samples was proved to be significantly different between the [normal + TMAO] and normal group.

## Materials and Methods

### Materials

Unless otherwise specified, all chemicals used in this study were commercially available. TMAO·2H_2_O and *p*-dimethylaminobenzonitrile (DMABN) were purchased from Yuanye Bio-Technology Co., Ltd. (Shanghai, China). Sodium chloride, *α*-cyclodextrins (*α*-CD), *β*-cyclodextrins (*β*-CD), *γ*-cyclodextrins (*γ*- CD), cucurbit[6]uril hydrate (CB6), cucurbit[7]uril hydrate (CB7), cucurbit[8]uril hydrate (CB8), 2-[4- (2-hydroxyethyl)piperazin-1-yl]ethanesulfonic acid (HEPES) and deuterium oxide (D_2_O) were obtained from Sigma-Aldrich Co., Ltd. (St. Louis, Missouri, USA). Fluorescein (Fl), 8-hydroxypyrene-1,3,6-trisulfonate (HPTS), acridine orange (AO), 2-(*p*-toluidinyl)naphthalene-6-sulfonic acid (2,6-TNS), glutamic acid (Glu), aspartic acid (Asp) and fumaric acid were manufactured by Tokyo Chemical Industry Co., Ltd. (Tokyo, Japan). Lucigenin (LCG), urea, potassium chloride and sodium phosphate (monobasic) were provided by Aladdin Co., Ltd. (California, USA). *Trans*-4-[4-(dimethylamino)styryl]-1-methylpyridinium iodide (DSMI) was purchased from Tianjin Heowns Biochemical Technology Co., Ltd. (Tianjin, China). Bovine serum albumin (BSA) and creatinine were purchased from J&K Chemical Co., Ltd (Beijing, China). *N,N'*-dimethyl-2,7-diazapyrenium (Me_2_DAP) was given as a gift from Prof. Frank Biedermann from Institute of Nanotechnology, Karlsruhe Institute of Technology (Eggenstein-Leopoldshafen, Germany). GC5A, *p*-sulfonatocalix[4]arenes (SC4A), *p*-sulfonatocalix[5]arenes (SC5A) and *p*-sulfonatocalix[6]arenes (SC6A) were synthesized as previously reported 26, 42-44.

### Preparation of HEPES buffer solution

HEPES buffer solution (10 mM, pH 7.4) was obtained by dissolving 2.38 g HEPES in approximately 900 mL ultra-pure water. The solution was titrated with sodium hydroxide to pH 7.4 at 25 °C, which was then diluted to 1 L with ultra-pure water to make a 10 mM HEPES buffer solution. The pH value of the buffer solution was checked with a Sartorius PB-20 pH-meter adjusted by three standard buffer solutions.

### Preparation of artificial urine solution

Artificial urine solution was obtained as previously reported 45, which was briefly prepared by dissolving urea (36.40 g), sodium chloride (15.00 g), sodium phosphate (monobasic, 9.60 g), potassium chloride (9.00 g), creatinine (4.00 g) and BSA (100 mg) in 2 L ultra-pure water. The artificial urine solution was verified to pH 6.0 with hydrochloric acid or sodium hydroxide and stored at 4 ºC.

### Preparation of urine samples

Written informed consent was signed by five healthy volunteers (22-33 years old), who provided urine samples maintained at -20 ºC until analysis. The supernatant of urine samples was collected after 10 min centrifugation (14000 rpm) and diluted twofold with 10 mM HEPES buffer solution (pH 7.4) before analysis.

### Fluorescence spectroscopy

The steady-state fluorescence spectra were recorded in a conventional 10 × 10 × 45 mm^3^ quartz cell (light path = 10 mm) by Varian Cary Eclipse spectrometer (Agilent Technologies Inc., USA). In order to eliminate the affect on the measured result caused by temperature variation, a Varian Cary single-cell Peltier accessory was equipped.

The excitation wavelengths for Fl, DMABN, 2,6-TNS, HPTS, LCG, DSMI, AO, and Me_2_DAP were 500, 300, 350, 405, 368, 450, 450 and 335 nm, respectively 38. For the direct host-guest titrations at 25 ºC in 10 mM HEPES buffer solution (pH 7.4), the dye solutions (0.50 - 10.00 μM) were excited at their respect excitation wavelengths to afford a emission, and the sequential changes of fluorescence intensity at various concentrations of hosts were used to determine the host-guest association constant (*K*_a_). The data were fitted according to a 1:1 host-guest (with the exception of 1:2 for SC6A•LCG) binding stoichiometry 39, 46, 47.

The competive fluorescence titrations in 10 mM HEPES buffer solution (pH 7.4) at 25 ºC were carried out by gradual addition of competitor (TMAO) with known concentration to solutions containing hosts and dyes with appropriate concentrations, which gave rise to variation of the fluorescence intensities of dyes. The association constants of TMAO with hosts were obtained by fitting fluorescence intensities at their emission wavelengths (Fl: 513 nm, LCG: 505 nm) and TMAO cocentrations according to the 1:1 competitive binding model 48. In the course of the titrations, the concentration of hosts and dyes were kept constant.

The calibration line was established at 25 °C according to the fluorescence intensities of GC5A•Fl reporter pair by gradual addition of TMAO with known concentrations in HEPES buffer (10 mM, pH 7.4) and artificial urine solution, respectively.

### NMR spectroscopy

^1^H NMR spectra were recorded on a Bruker AV400 spectrometer (Bruker Group, Switzerland) using fumaric acid (*δ* = 6.70 ppm) as an external reference. 2D ROESY spectrum was recorded on a Zhongke-Niujin BIXI-I 400 spectrometer (Zhongke-Niujin, Wuhan, China). Samples of GC5A•TMAO complex, GC5A and TMAO for NMR measurement were prepared in D_2_O. All NMR spectra were acquired at 298 K in the solution state.

### Theoretical Calculations

A Gaussian 09 program density was employed to perform functional theory calculations 49. Based on density, the solvation model was undertaken in all calculations 50. At the B3LYP/6-31G(d) level, geometry optimization was carried out by using Grimme's D3 dispersion correction 51, 52. Default convergence criteria were used for the optimization in Gaussian 09.

### Data analysis

Mean values of fluorescence titrations, calibration lines and limit of detection were determined from at least three experiments, and errors were given as standard deviations (± SD).

The fitting of data from direct host-guest titrations and competitive titrations were performed in a nonlinear manner 39, and the fitting modules were downloaded from the website of Prof. Nau's group (http://www.jacobs-university.de/ses/wnau) under the column of “Fitting Functions”. It was introduced in detail as following.

For analyzing the host-guest fluorescence titrations as described by equation 1, we considered that a guest (G) formed a 1:1 host•guest complex with a host (H) at an association constant (*K*_a_), which satisfied the respective law of mass action relating to the equilibrium concentrations of free host, [H], free guest, [G], and host•guest complex [HG]. Also, the relationship between the total concentrations of host, [H]_0_, and guest, [G]_0_, and their equilibrium concentrations were introduced by the law of mass conservation (equation 2). Here, [G]_0_ was the initial concentration of guest as a known experimental parameter, which was kept constant in the titration process. Furthermore, equation 1 and 2-1 were employed to deduce equation 3.

When the fluorescence titration was performed, the intensity of fluorescence (*F*) corresponded to the combined intensity of the guest and the host•guest complex, which were described by their molar fractions (equation 4). Both *F*_HG_ and* F*_G_ were the known experimental parameters, in which *F*_HG_ was the fluorescence intensity when all guests were complexed and *F*_G_ when they were uncomplexed. The equation 5 deduced by equation 2-2, 3 and 4, explained the relationship between *K*_a_ and variables ([H]_0_) in fluorescence titration 47. In the light of equation 5, *K*_a_ was obtained by fitting the data of fluorescence intensity and total host concentration.


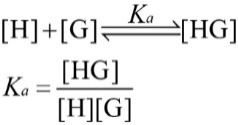
(1)


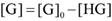
(2-1)


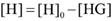
(2-2)


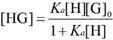
(3)


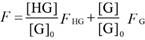
(4)


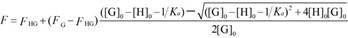
(5)

For the analysis of the competitive titrations (equation 6), we also considered a competitor (TMAO) that could bind to a host's cavity in a 1:1 stoichiometry at an association constant (*K*_T_). Free host, [H], free competitor, [T], and host•TMAO complex [HT] obeyed the respective law of mass action referring to the equilibrium concentrations. Also, [H]_0_ and the total concentrations of TMAO, [T]_0_, and their equilibrium concentrations satisfied the law of mass conservation (equation 7).

In the course of the titration, the fluorescence intensity (*F*_C_) was expressed as a linear combination of *F*_HG_ and* F*_G_, weighted by their molar fractions on the basis of equation 8. Through a 1:1 host•guest binding model, *F*_HG_ was further denoted 47 by the (initial) experimental fluorescence intensity in the absence of TMAO. Substituting equation 3 into equation 8 gave equation 9, with the concentration of uncomplexed host as an unknown parameter, [H], which was numerically solved by a cubic equation (equation 10) with Newton-Raphson algorithm 53, 54. In addition, equation 10 was deduced by combining equation 3, 6, 7-1 and 7-2. For fitting, the fluorescence intensity was plotted against [T]_0_ based on equation 9 in a program.


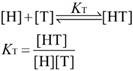
(6)


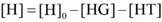
(7-1)


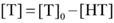
(7-2)


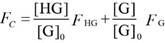
(8)


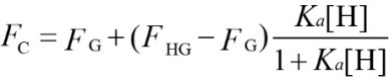
(9)


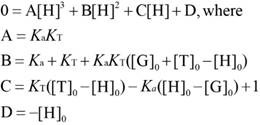
(10)

## Results and Discussion

### Screening of TMAO artificial receptors from classical water-soluble macrocycles

**Scheme 1:**
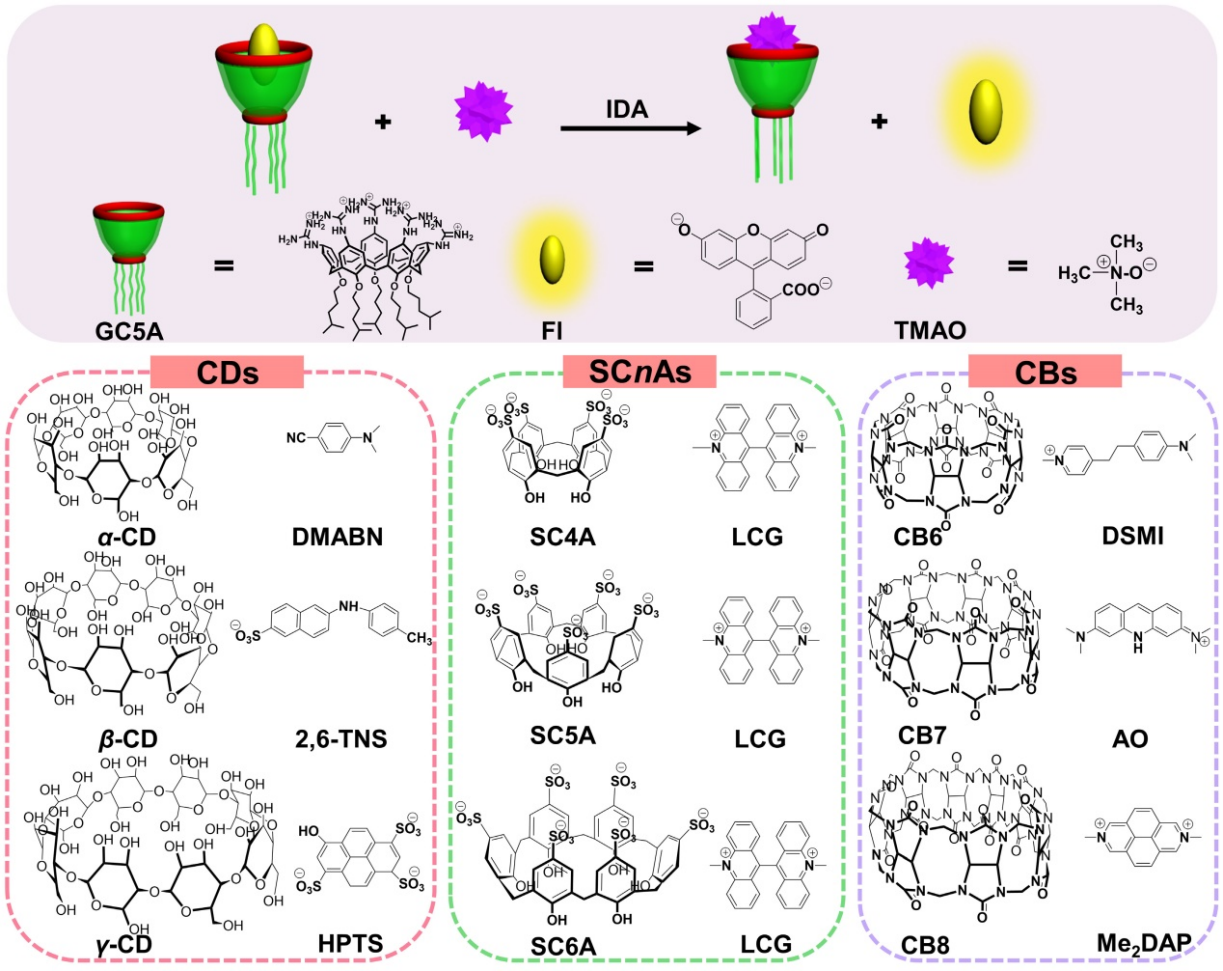
Schematic illustration for IDA operating principle of TMAO fluorescence “switch-on” sensing by the GC5A•Fl reporter pair and chemical structures of the tested reporter pairs.

IDA (Scheme 1) was popularized by Anslyn and co-workers, the synthetic receptors with different styles were employed for molecular sensing via competitive binding assays 20. In this classical model, introduction of a competitive analyte causes the shift of fluorescence intensity due to extrusion of fluorescence indicator from the host, which feasibly paved the way for determining analytes without chromophores or chemical labeling. Importantly, the sifting of host and indicator with the appropriate binding affinity must be accomplished, allowing for the linear fluorescence response of indicator along with increase of analyte concentration. Therefore, the key step to develop a successful IDA for TMAO is to screen artificial receptors with strong and selective binding towards TMAO.

The most popular water-soluble macrocyclic receptors commercially available 46, including cyclodextrins (*α*-, *β*-, and *γ*-CD), *p*-sulfonatocalix[*n*]-arenes (SC*n*As, *n* = 4, 5, 6) and cucurbit[*n*]urils (CB*n*, *n* = 6, 7, 8), were selected for sensing and quantification of TMAO. As for the CD family, the fluorescence response was not influenced by the gradual addition of TMAO after screening of three reporter pairs, *α*-CD•DMABN 55, *β*-CD•2,6-TNS 56 and *γ*-CD• HPTS 57 (Figures S1 - S3, Table 1). Unfortunately, similar result happened to the SC*n*A family except for SC4A (Figures S4 - S6, Table 1). LCG showed slight response by gradual addition of TMAO to the SC4A•LCG reporter pair 38, 39, 46, which arose from fluorescence enhancement in view of LCG extrusion from SC4A. The binding affinity of SC4A towards TMAO was 65.51 ± 3.61 M^-1^, which cannot satisfy the implementation of IDA. Benefiting from extremely strong binding capability, the CB family came into our sight, with the highest binding affinity of 7.2 × 10^17^ M^-1^ between CB7 and diamantane quaternary diammonium ion 58. Therefore, the feasibility of TMAO quantification was demonstrated by employing CB6•DSMI 59, CB7•AO 60 and CB8•Me_2_DAP 61. However, the binding towards TMAO was not obtained by the CB family using the competitive fluorescence titrations (Figures S7 - S9, Table 1). In general, failure of TMAO quantification was due to the unsatisfactory non-covalent interactions, such as hydrogen bonding, hydrophobic interaction and van der Waals forces, between host and guest. In our opinion, the hydrophobic cavities of CDs and CBs families cannot afford the ideal binding affinity with water-soluble TMAO 32. SC*n*As with negative charge cannot successfully encapsulate TMAO, a neutral polar compound 62. This study revealed that the molecular recognition of TMAO by artificial receptors posed a challenge to us. It drove us to search for the ideal reporter pair for TMAO quantification.

### The study on formation of the GC5A•TMAO complex

**Figure 1:**
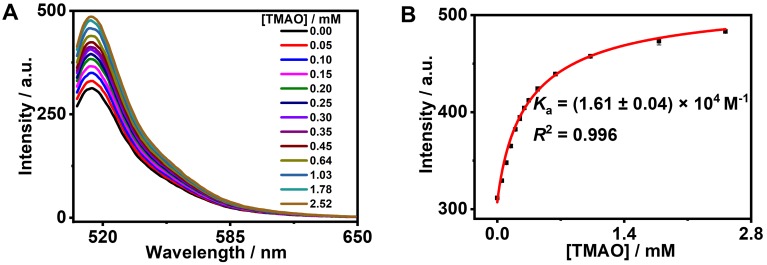
Fluorescence competitive titration in the GC5A•Fl (0.80/1.00 μM) reporter pair with TMAO (up to 2.52 mM) at *λ*_ex_ = 500 nm (A) and competitive titration curve (*λ*_em_ = 513 nm) and fitting data according to a 1:1 competitive binding model (B) in HEPES buffer solution (10 mM, pH 7.4) at 25 °C.

GC5A, a new water-soluble calixarene, was synthesized and first applied in detecting lysophosphatidic acid in blood as a biomarker of cancer via fluorescence IDA by our group 26. Also, GC5A was successfully employed in identifying six glycosaminoglycans 25. Furthermore, the *K*_a_ ((5.0 ± 1.0) × 10^6^ M^-1^) of GC5A•Fl was validated by the fluorescence titration and UV-Vis titration 26. Reasonably, GC5A-based molecular recognition and sensing were performed to determine TMAO with the aid of Fl as the indicator for IDA 26. Importantly, the fluorescence intensity of Fl was not affected by gradual addition of TMAO (0 - 9.48 mM) (Figure S10). As shown in Figure 1, the displacement of Fl with TMAO from the GC5A•Fl reporter pair was monitored by the increase in fluorescence signal. Fortunately, GC5A afforded a relatively strong binding affinity ((1.61 ± 0.04) × 10^4^ M^-1^) towards TMAO, which shed light on the puzzling question for TMAO quantification.

**Figure 2:**
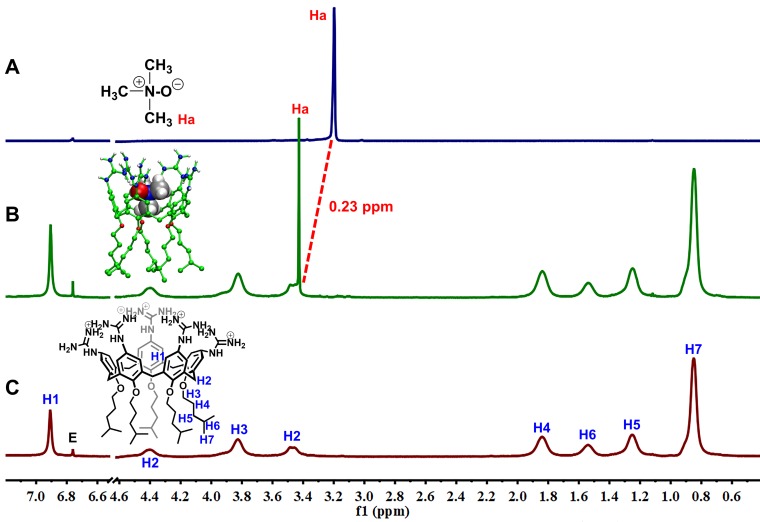
^1^H NMR spectra (400 MHz, 298 K) of TMAO (2.00 mM) (A), TMAO (0.40 mM) with addition of GC5A (2.00 mM) (B) and GC5A (2.00 mM) (C) using fumaric acid (peak E in NMR spectra) as an external reference in D_2_O. The optimized structure of the GC5A•TMAO complex at the B3LYPD3(BJ)/6-31G(d)/SMD (water) level of theory shown in the inset of Figure 2B. Some hydrogen atoms were omitted for clarity.

The encapsulation of TMAO into GC5A resulted in forming the GC5A•TMAO complex, which was validated by ^1^H NMR spectroscopy (Figure 2). As soon as TMAO has been titrated into GC5A, the signal assigned to TMAO methyl protons shifted downfield (Δ*δ* = 0.23 ppm). The shift of TMAO protons were possibly caused by the intermolecular hydrogen bonding and ring currents effect, which resulted in shift to downfield and upfield, respectively 63-65. Compared to the negatively charged or uncharged calixarenes 39, 63, 66, the GC5A with positively charged substituents had the lower electrostatic potential, leading to a relatively weak ring currents effect 26. Therefore, the shift was dominated by the intermolecular hydrogen bonding, which was lessened under the influence of the aromatic ring currents of GC5A. The signals of the protons from GC5A did not shift appreciably, because TMAO lacked groups (such as aromatic rings) that could result in significant shielding or deshielding. The assumed binding geometry was further demonstrated by the 2D ROESY spectrum (Figure S11). The intermolecular correlation was observed between the methyl protons of TMAO and aromatic protons of GC5A. Geometry optimization of the GC5A•TMAO complex revealed the formation of hydrogen bonding interaction and the size/shape matching between the GC5A cavity and TMAO (Figure 2B inset), which was consistent with the NMR information 51, 67-69.

### Quantification of TMAO by the GC5A•Fl reporter pair

**Figure 3:**
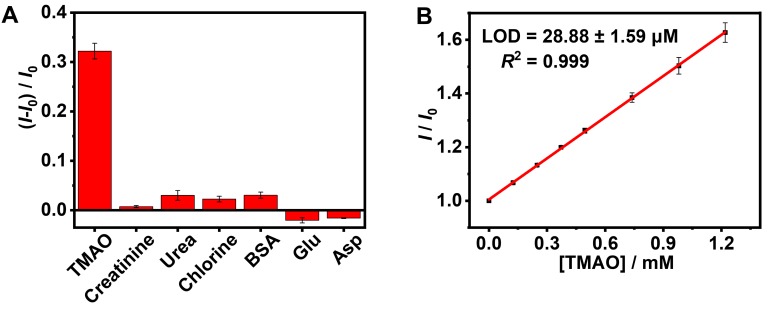
Fluorescence response in the GC5A•Fl (0.80/1.00 μM) reporter pair at 513 nm (*λ*_ex_ = 500 nm) upon addition of TMAO and potentially interfering substances from artificial urine (0.40 mg/L for BSA and 0.30 mM for other interfering substances, respectively) in HEPES buffer solution (10 mM, pH 7.4) at 25 °C (A). The calibration line of fluorescence intensity for quantitatively determining TMAO in artificial urine at 25 °C (B). *I* and *I*_0_ were the fluorescence intensities of the GC5A•Fl reporter pair in the presence and absence of analyte. Error bars smaller than 0.005 were not shown.

As shown in Figure S12, the elevated TMAO concentration was accompanied by an increased fluorescence signal (*R*^2^ = 0.980), which supported fluorescence “switch-on” sensing of TMAO in the GC5A•Fl (0.80/1.00 µM) reporter pair. The limit of detection (LOD) of TMAO was achieved at 8.98 ± 0.06 µM in the HEPES buffer solution using the 3*σ*/slope method 70, 71. Subsequently, the feasibility of TMAO quantification was tested in artificial urine. It was very important to verify whether the matrix substances posed interference or not, including creatinine, urea, chlorine, bovine serum albumin (BSA), glutamic acid (Glu) and aspartic acid (Asp) (Figure 3A), conducing to evaluating the sensing selectivity of GC5A•Fl reporter pair to TMAO. No obvious interference was detected upon addition of matrix substances into the GC5A•Fl reporter pair.

**Figure 4:**
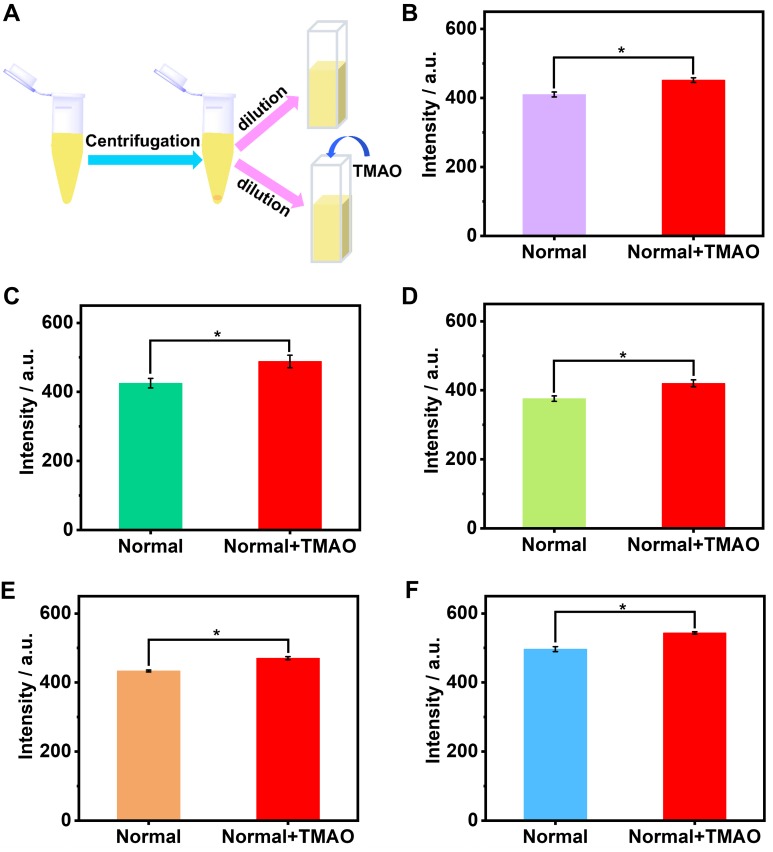
The preprocessing procedure of urine samples (A). Fluorescence response of GC5A•Fl reporter pair (10.00 / 5.00 μM) at 513 nm (*λ*_ex_ = 500 nm) from the twofold diluted urine samples in the presence and absence of the added TMAO (0.1 mM) from five healthy volunteers (B - F). Significance was measured with Student's t test. *p < 0.05. Error bars represented the standard derivations of three independent determination.

The artificial urine was employed to verify application of GC5A•Fl in TMAO quantification. Accompanied by the increased TMAO concentration, the elevated fluorescence signal was linearly monitored in artificial urine in the range of 0 to 1.22 mM (*R*^2^ = 0.999, Figure 3B), which also demonstrated that the interference from the matrix of artificial urine could be excluded in the GC5A•Fl (10.00/5.00 μM) reporter pair. The TMAO quantification was proved to be sensitive by obtaining LOD low to 28.88 ± 1.59 µM in artificial urine. Furthermore, the GC5A•Fl reporter pair was successfully applied in analyzing TMAO in authentic human urine samples from the healthy volunteers. According to the reported TMAO concentration (approximately 0.20 mM) in urine from infarcted patients 72, the corresponding TMAO was added to the samples of normal group. As shown in Figure 4, the significant difference in fluorescence response was identified between the [normal + TMAO] and normal group.

In general, we herein achieved sensitive detection of TMAO, arising from the binding capability of GC5A towards TMAO as well as the remarkable fluorescence regeneration of Fl from the GC5A•Fl reporter pair. A calibration line was set up for accurately determining TMAO concentration down to the low µM range, based on the linear relationship between the fluorescence intensity and TMAO concentration, principally conducing to track TMAO excreted in urine. Also, a successful application was performed in human urine samples to distinctly identify the difference of fluorescence response between the [normal + TMAO] and normal group.

## Conclusions

In conclusion, we have established an IDA method for the fluorescence “switch-on” sensing and quantitative assay of TMAO in artificial urine via the GC5A•Fl reporter pair. To accurately determine concentrations of TMAO in the low μM range for practical diagnostic purpose, a calibration line of TMAO in artificial urine was successfully established. The feasibility was approved by application in detecting TMAO in authentic human urine samples from the healthy volunteers. In comparison to the previous methods, this study provides a low-cost, easy-to-operate, label-free and sensitive method for detecting TMAO, which may offer an alternative for TMAO detection in the clinical study. This method shows promise for application in tracking TMAO excretion and studying chronic disease progression in humans.

## Supplementary Material

Supplementary figures and tables.

## Figures and Tables

**Table 1:** The reporter pairs of fluorescent dyes and macrocycles utilized for detecting TMAO by IDA.

Reporter pair		*K*_a_ (dye) / M^-1^	*λ*_ex_ (*λ*_em_) ^a^ / nm	*K*_a_ (TMAO) / M^-1 b, c^
macrocycle	dye			
*α*-CD	DMABN	(2.58 ± 0.03) × 10^2^	300 (525)	-
*β*-CD	2,6-TNS	(1.88 ± 0.36) × 10^3^	350 (483)	-
*γ*-CD	HPTS	(5.61 ± 0.28) × 10^1^	405 (435)	-
SC4A	LCG	(1.26 ± 0.13) × 10^7^	368 (505)	(6.55 ± 0.36) × 10^1^
SC5A	LCG	(1.48 ± 0.21) × 10^6^	368 (505)	-
SC6A^d^	LCG	(4.96 ± 0.62) × 10^7^	368 (505)	-
CB6	DSMI	(1.68 ± 0.26) × 10^5^	450 (582)	-
CB7	AO	(9.04 ± 0.54) × 10^4^	450 (510)	-
CB8	Me_2_DAP	(1.27 ± 0.42) × 10^6^	335 (449)	-


^a^ *λ*_ex_ represents the fluorescence excitation wavelength.* λ*_em_ represents the maximum fluorescence emission wavelength. ^b^ represented the binding affinity of macrocycle towards TMAO. ^c^ “-” represented no binding detected. ^d^ data fit with the host:guest 1:2 binding model.

## Abbreviations

AO: acridine orange
Asp: aspartic acid
BSA: bovine serum albumin
CB: cucurbituril
CD: cyclodextrin
DMABN: *p*-dimethylaminobenzonitrile
D_2_O: deuterium oxide
DSMI: *trans*-4-[4-(dimethylamino)-styryl]-1-methylpyridinium iodide
2D ROESY: two dimensional rotating‐frame nuclear Overhauser effect spectroscopy
Fl: fluorescein
GC5A: guanidinium- modified calix[5]arene
GC-MS: gas chromatography- mass spectrometry
Glu: glutamic acid
^1^H NMR: proton nuclear magnetic resonance
HEPES: 2-[4- (2-hydroxyethyl)piperazin-1-yl]ethanesulfonic acid
HPTS: 8-hydroxypyrene-1,3,6-trisulfonate
IDA: indicator displacement assay
LC-MS/MS: liquid chromatography tandem mass spectrometry
LCG: lucigenin
LOD: limit of detection
Me_2_DAP: *N,N'*-dimethyl-2,7-diazapyrenium
SC*n*A: *p*-sulfonatocalix[*n*]arene
SD: standard deviations
TMA: trimethylamine
TMAO: trimethylamine *N*-oxide
2,6-TNS: 2-(*p*-toluidinyl)-naphthalene-6-sulfonic acid

## References

[1] Wang Z, Klipfell E, Bennett BJ, Koeth R, Levison BS, DuGar B (2011). Gut flora metabolism of phosphatidylcholine promotes cardiovascular disease. Nature.

[2] Koeth RA, Wang Z, Levison BS, Buffa JA, Org E, Sheehy BT (2013). Intestinal microbiota metabolism of *L*-carnitine, a nutrient in red meat, promotes atherosclerosis. Nat Med.

[3] Zhu W, Gregory JC, Org E, Buffa JA, Gupta N, Wang Z (2016). Gut microbial metabolite TMAO enhances platelet hyperreactivity and thrombosis risk. Cell.

[4] Missailidis C, Hällqvist J, Qureshi AR, Barany P, Heimbürger O, Lindholm B (2016). Serum trimethylamine-*N*-oxide is strongly related to renal function and predicts outcome in chronic kidney disease. PLoS One.

[5] Shan Z, Sun T, Huang H, Chen S, Chen L, Luo C (2017). Association between microbiota-dependent metabolite trimethylamine-*N*-oxide and type 2 diabetes. Am J Clin Nutr.

[6] Chen Y-M, Liu Y, Zhou R-F, Chen X-L, Wang C, Tan X-Y (2016). Associations of gut-flora-dependent metabolite trimethylamine-*N*-oxide, betaine and choline with non-alcoholic fatty liver disease in adults. Sci Rep.

[7] Bae S, Ulrich CM, Neuhouser ML, Malysheva O, Bailey LB, Xiao L (2014). Plasma choline metabolites and colorectal cancer risk in the women's health initiative observational study. Cancer Res.

[8] Zeisel SH, Warrier M (2017). Trimethylamine *N*-oxide, the microbiome, and heart and kidney disease. Annu Rev Nutr.

[9] Bain MA, Faull R, Fornasini G, Milne RW, Evans AM (2006). Accumulation of trimethylamine and trimethylamine-*N*-oxide in end-stage renal disease patients undergoing haemodialysis. Nephrol Dial Transplant.

[10] Wang Z, Levison BS, Hazen JE, Donahue L, Li XM, Hazen SL (2014). Measurement of trimethylamine-*N*-oxide by stable isotope dilution liquid chromatography tandem mass spectrometry. Anal Biochem.

[11] Ocque AJ, Stubbs JR, Nolin TD (2015). Development and validation of a simple UHPLC-MS/MS method for the simultaneous determination of trimethylamine *N*-oxide, choline, and betaine in human plasma and urine. J Pharm Biomed Anal.

[12] Romano KA, Vivas EI, Amador-Noguez D, Rey FE (2015). Intestinal microbiota composition modulates choline bioavailability from diet and accumulation of the proatherogenic metabolite trimethylamine-*N*-oxide. MBio.

[13] Zhao X, Zeisel SH, Zhang S (2015). Rapid LC-MRM-MS assay for simultaneous quantification of choline, betaine, trimethylamine, trimethylamine *N*-oxide, and creatinine in human plasma and urine. Electrophoresis.

[14] Heaney LM, Jones DJ, Mbasu RJ, Ng LL, Suzuki T (2016). High mass accuracy assay for trimethylamine *N*-oxide using stable-isotope dilution with liquid chromatography coupled to orthogonal acceleration time of flight mass spectrometry with multiple reaction monitoring. Anal Bioanal Chem.

[15] Liu J, Zhao M, Zhou J, Liu C, Zheng L, Yin Y (2016). Simultaneous targeted analysis of trimethylamine-*N*-oxide, choline, betaine, and carnitine by high performance liquid chromatography tandem mass spectrometry. J Chromatogr B Analyt Technol Biomed Life Sci.

[16] Steuer C, Schütz P, Bernasconi L, Huber AR (2016). Simultaneous determination of phosphatidylcholine-derived quaternary ammonium compounds by a LC-MS/MS method in human blood plasma, serum and urine samples. J Chromatogr B Analyt Technol Biomed Life Sci.

[17] Lee MB, Storer MK, Blunt JW, Lever M (2006). Validation of ^1^H NMR spectroscopy as an analytical tool for methylamine metabolites in urine. Clin Chim Acta.

[18] Podadera P, Sipahi AM, Arêas JA, Lanfer-Marquez UM (2005). Diagnosis of suspected trimethylaminuria by NMR spectroscopy. Clin Chim Acta.

[19] Garcia E, Wolak-Dinsmore J, Wang Z, Li XS, Bennett DW, Connelly MA (2017). NMR quantification of trimethylamine-*N*-oxide in human serum and plasma in the clinical laboratory setting. Clin Biochem.

[20] Nguyen BT, Anslyn EV (2006). Indicator-displacement assays. Coordin Chem Rev.

[21] Ghale G, Nau WM (2014). Dynamically analyte-responsive macrocyclic host-fluorophore systems. Acc Chem Res.

[22] Lee HH, Choi TS, Lee SJC, Lee JW, Park J, Ko YH (2014). Supramolecular inhibition of amyloid fibrillation by cucurbit[7]uril. Angew Chem Int Ed.

[23] Shinde MN, Barooah N, Bhasikuttan AC, Mohanty J (2016). Inhibition and disintegration of insulin amyloid fibrils: a facile supramolecular strategy with *p*-sulfonatocalixarenes. Chem Commun.

[24] Gao J, Li J, Geng W-C, Chen F-Y, Duan X, Zheng Z (2018). Biomarker displacement activation: a general host-guest strategy for targeted phototheranostics *in vivo*. J Am Chem Soc.

[25] Zheng Z, Geng W-C, Gao J, Mu Y-J, Guo D-S (2018). Differential calixarene receptors create patterns that discriminate glycosaminoglycans. Org Chem Fron.

[26] Zheng Z, Geng W-C, Gao J, Wang Y-Y, Sun H, Guo D-S (2018). Ultrasensitive and specific fluorescence detection of a cancer biomarker via nanomolar binding to a guanidinium-modified calixarene. Chem Sci.

[27] Yu G, Zhao X, Zhou J, Mao Z, Huang X, Wang Z (2018). Supramolecular polymer-based nanomedicine: high therapeutic performance and negligible long-term immunotoxicity. J Am Chem Soc.

[28] Yu G, Yang Z, Fu X, Yung BC, Yang J, Mao Z (2018). Polyrotaxane-based supramolecular theranostics. Nat Commun.

[29] Zhou J, Yu G, Huang F (2017). Supramolecular chemotherapy based on host-guest molecular recognition: a novel strategy in the battle against cancer with a bright future. Chem Soc Rev.

[30] Webber MJ, Langer R (2017). Drug delivery by supramolecular design. Chem Soc Rev.

[31] Ma D, Hettiarachchi G, Nguyen D, Zhang B, Wittenberg JB, Zavalij PY (2012). Acyclic cucurbit[*n*]uril molecular containers enhance the solubility and bioactivity of poorly soluble pharmaceuticals. Nat Chem.

[32] Ghosh I, Nau WM (2012). The strategic use of supramolecular p*K*_a_ shifts to enhance the bioavailability of drugs. Adv Drug Deliv Rev.

[33] Li B, Meng Z, Li Q, Huang X, Kang Z, Dong H (2017). A pH responsive complexation-based drug delivery system for oxaliplatin. Chem Sci.

[34] Lazar AI, Biedermann F, Mustafina KR, Assaf KI, Hennig A, Nau WM (2016). Nanomolar binding of steroids to cucurbit[*n*]urils: selectivity and applications. J Am Chem Soc.

[35] Daze KD, Pinter T, Beshara CS, Ibraheem A, Minaker SA, Ma MCF (2012). Supramolecular hosts that recognize methyllysines and disrupt the interaction between a modified histone tail and its epigenetic reader protein. Chem Sci.

[36] McGovern RE, Fernandes H, Khan AR, Power NP, Crowley PB (2012). Protein camouflage in cytochrome *c*-calixarene complexes. Nat Chem.

[37] Guo D-S, Liu Y (2014). Supramolecular chemistry of *p*-sulfonatocalix[*n*]arenes and its biological applications. Acc Chem Res.

[38] Guo D-S, Yang J, Liu Y (2013). Specifically monitoring butyrylcholinesterase by supramolecular tandem assay. Chem - Eur J.

[39] Guo D-S, Uzunova VD, Su X, Liu Y, Nau WM (2011). Operational calixarene-based fluorescent sensing systems for choline and acetylcholine and their application to enzymatic reactions. Chem Sci.

[40] Guo D-S, Wang K, Wang Y-X, Liu Y (2012). Cholinesterase-responsive supramolecular vesicle. J Am Chem Soc.

[41] Gao J, Zheng Z, Shi L, Wu S-Q, Sun H, Guo D-S (2018). Strong binding and fluorescence sensing of bisphosphonates by guanidinium-modified calix[5]arene. Beilstein J Org Chem.

[42] Shinkai S, Mori S, Tsubaki T, Sone T, Manabe O (1984). New water-soluble host molecules derived from calix[6]arene. Tetrahedron Lett.

[43] Shinkai S, Mori S, Koreishi H, Tsubaki T, Manabe O (1986). Hexasulfonated calix[6]arene derivatives: a new class of catalysts, surfactants, and host molecules. J Am Chem Soc.

[44] Steed JW, Johnson CP, Barnes C, Juneja RK, Atwood JL, Reilly S (1995). Supramolecular chemistry of *p*-sulfonatocalix[5]arene: a water-soluble, bowl-shaped host with a large molecular cavity. J Am Chem Soc.

[45] Shmaefsky BR (1990). Artificial Urine for Laboratory Testing. Am Biol Teach.

[46] Dsouza RN, Pischel U, Nau WM (2011). Fluorescent dyes and their supramolecular host/guest complexes with macrocycles in aqueous solution. Chem Rev.

[47] Nau WM, Zhang X (1999). An exceedingly long-lived fluorescent state as a distinct structural and dynamic probe for supramolecular association: an exploratory study of host-guest complexation by cyclodextrins. J Am Chem Soc.

[48] Bakirci H, Koner AL, Nau WM (2005). Binding of inorganic cations by *p*-sulfonatocalix[4]arene monitored through competitive fluorophore displacement in aqueous solution.

[49] Frisch MJ, Trucks GW, Schlegel HB, Scuseria GE, Robb MA, Cheeseman JR Gaussian 09, Revision E.01, Gaussian, Inc, Wallingford CT, 2013.

[50] Marenich AV, Cramer CJ, Truhlar DG (2009). Universal solvation model based on solute electron density and on a continuum model of the solvent defined by the bulk dielectric constant and atomic surface tensions. J Phys Chem B.

[51] Stephens PJ, Devlin FJ, Chabalowski CF, Frisch MJ (1994). Ab initio calculation of vibrational absorption and circular dichroism spectra using density functional force fields. J Phys Chem.

[52] Grimme S, Antony J, Ehrlich S, Krieg H (2010). A consistent and accurate ab initio parametrization of density functional dispersion correction (DFT-D) for the 94 elements H-Pu. J Chem Phys.

[53] Bakirci H, Nau WM (2006). Fluorescence regeneration as a signaling principle for choline and carnitine binding: a refined supramolecular sensor system based on a fluorescent azoalkane. Adv Funct Mater.

[54] Hennig A, Bakirci H, Nau WM (2007). Label-free continuous enzyme assays with macrocycle-fluorescent dye complexes. Nat Methods.

[55] Shaikh M, Mohanty J, Bhasikuttan AC, Pal H (2008). Tuning dual emission behavior of *p*-dialkylaminobenzonitriles by supramolecular interactions with cyclodextrin hosts. Photochem Photobiol Sci.

[56] Liu Y, You C-C (2001). Inclusion complexation of *β*-cyclodextrin and 6-*O*-*α*-maltosyl- and 2-*O*-(2-hydroxypropyl)-*β*-cyclodextrins with some fuorescent dyes. J Phys Org Chem.

[57] Mondal SK, Sahu K, Sen P, Roy D, Ghosh S, Bhattacharyya K (2005). Excited state proton transfer of pyranine in a *γ*-cyclodextrin cavity. Chem Phys Lett.

[58] Cao L, Šekutor M, Zavalij PY, Mlinarić-Majerski K, Glaser R, Isaacs L (2014). Cucurbit[7]uril•guest pair with an attomolar dissociation constant. Angew Chem Int Ed.

[59] Li Z, Sun S, Liu F, Pang Y, Fan J, Song F (2012). Large fluorescence enhancement of a hemicyanine by supramolecular interaction with cucurbit[6]uril and its application as resettable logic gates. Dye Pig.

[60] Nau WM, Ghale G, Hennig A, Bakirci H, Bailey DM (2009). Substrate-selective supramolecular tandem assays: monitoring enzyme inhibition of arginase and diamine oxidase by fluorescent dye displacement from calixarene and cucurbituril macrocycles. J Am Chem Soc.

[61] Sindelar V, Cejas MA, Raymo FM, Chen W, Parker SE, Kaifer AE (2005). Supramolecular assembly of 2,7-dimethyldiazapyrenium and cucurbit[8]uril: a new fluorescent host for detection of catechol and dopamine. Chem - Eur J.

[62] Guo D-S, Wang K, Liu Y (2008). Selective binding behaviors of *p*-sulfonatocalixarenes in aqueous solution. J Incl Phenom Macrocycl Chem.

[63] De Rosa M, Talotta C, Gaeta C, Soriente A, Neri P, Pappalardo S (2017). Calix[5]arene through-the-annulus threading of dialkylammonium guests weakly paired to the TFPB anion. J Org Chem.

[64] Guo D-S, Zhang H-Q, Ding F, Liu Y (2012). Thermodynamic origins of selective binding affinity between *p*-sulfonatocalix[4,5]arenes with biguanidiniums. Org Biomol Chem.

[65] Gattuso G, Notti A, Pappalardo S, Parisi MF, Pisagatti I, Patanè S (2014). Encapsulation of monoamine neurotransmitters and trace amines by amphiphilic anionic calix[5]arene micelles. New J Chem.

[66] Zhao H-X, Guo D-S, Liu Y (2013). Binding behaviors of *p*-sulfonatocalix[4]arene with gemini guests. J Phys Chem B.

[67] Hehre WJ, Ditchfield R, Pople JA (1972). Self-consistent molecular orbital methods. XII. further extensions of gaussian-type basis sets for use in molecular orbital studies of organic molecules. J Chem Phys.

[68] Hariharan PC, Pople JA (1973). The influence of polarization functions on molecular orbital hydrogenation energies. Theoret chim Acta.

[69] Becke AD (1993). Density-functional thermochemistry. III. the role of exact exchange. J Chem Phys.

[70] Huang G-B, Wang S-H, Ke H, Yang L-P, Jiang W (2016). Selective recognition of highly hydrophilic molecules in water by endo-functionalized molecular tubes. J Am Chem Soc.

[71] MacDougall D, Crummett WB, Amore FJ, Cox GV, Crosby DG, Estes FL (1980). Guidelines for data acquisition and data quality evaluation in environmental chemistry. Anal Chem.

[72] Cieslarova Z, Magaldi M, Barros LA, do Lago CL, Oliveira DR, Fonseca FAH (2019). Capillary electrophoresis with dual diode array detection and tandem mass spectrometry to access cardiovascular biomarkers candidates in human urine: trimethylamine-*N*-oxide and L-carnitine. J Chromatogr A.

