# Supplementary material for: Facile Fluorescence Monitoring of Gut Microbial Metabolite Trimethylamine N-oxide via Molecular Recognition of Guanidinium-Modified Calixarene
Source: Theranostics. 2019 Jun 24;9(16):4624–32. doi: 10.7150/thno.33459 (PMC6643440; doi:10.7150/thno.33459)
Supplement: Supplementary file 1 — Supplementary figures and tables. [file thnov09p4624s1.pdf]

Supplementary Material

Facile fluorescence monitoring of gut microbial metabolite trimethylamine *N*-oxide via molecular recognition of guanidinium-modified calixarene

Huijuan Yu[1], Wen-Chao Geng[2], Zhe Zheng[2], Jie Gao[2], Dong-Sheng Guo[2✉], and Yuefei Wang[1✉]

1. Institute of Traditional Chinese Medicine, Tianjin University of Traditional Chinese Medicine, Tianjin 301617, China
2. College of Chemistry, Key Laboratory of Functional Polymer Materials (Ministry of Education), State Key Laboratory of Elemento-Organic Chemistry, Tianjin Key Laboratory of Biosensing and Molecular Recognition, Nankai University, Tianjin 300071, China

✉ Corresponding author: Dong-Sheng Guo, Prof., College of Chemistry, Nankai University No. 94 Weijin Road, Nankai District, Tianjin 300071, China. Phone: +86-22-23498949; E-mail dshguo@nankai.edu.cn; Yuefei Wang, Prof., Institute of Traditional Chinese Medicine, Tianjin University of Traditional Chinese Medicine, No. 10 Poyanghu Road, Jinghai District, Tianjin 301617, China. Phone: +86-22-59596366; E-mail wangyf0622@tjutcm.edu.cn

Contents

1. Direct fluorescence titration of dyes with hosts and competitive titration in the reporter pairs with TMAO in HEPES buffer solution..1

2. Direct fluorescence titration of Fl with TMAO..10

3. 2D ROESY spectrum of GC5A•TMAO...11

4. The limit of detection (LOD) for TMAO in HEPES buffer solution 12

1. Direct fluorescence titration of dyes with hosts and competitive titration in the reporter pairs with TMAO in HEPES buffer solution

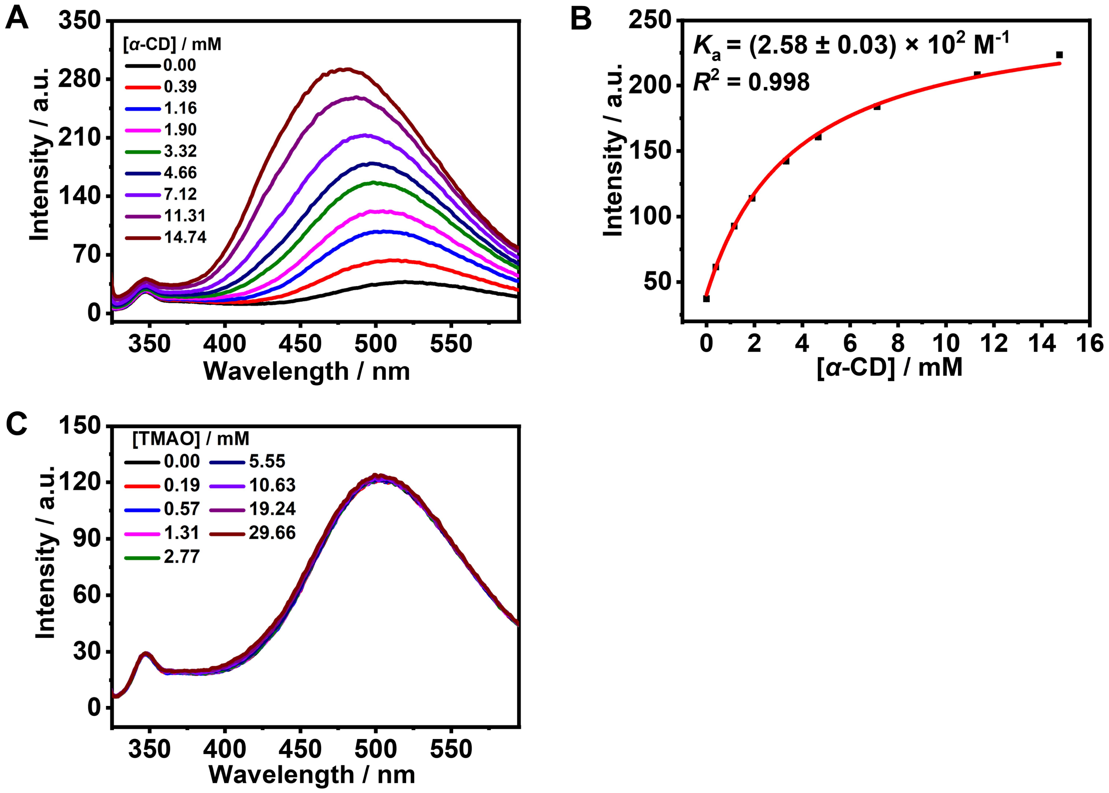

Figure S1. Direct fluorescence titration of DMABN (10.00 μM) with *α*-CD (up to 14.74 mM) at λ_{ex} = 300 nm (A), titration curve (λ_{em} = 525 nm) acquired by a 1:1 binding model (B), and competitive titration in the *α*-CD•DMABN (2.00 mM/10.00 μM) reporter pair with TMAO (up to 29.66 mM) at λ_{em} = 525 nm (C). All experiments were in HEPES buffer (10 mM, pH 7.4) at 25 °C. Error bars smaller than 0.005 were not shown.

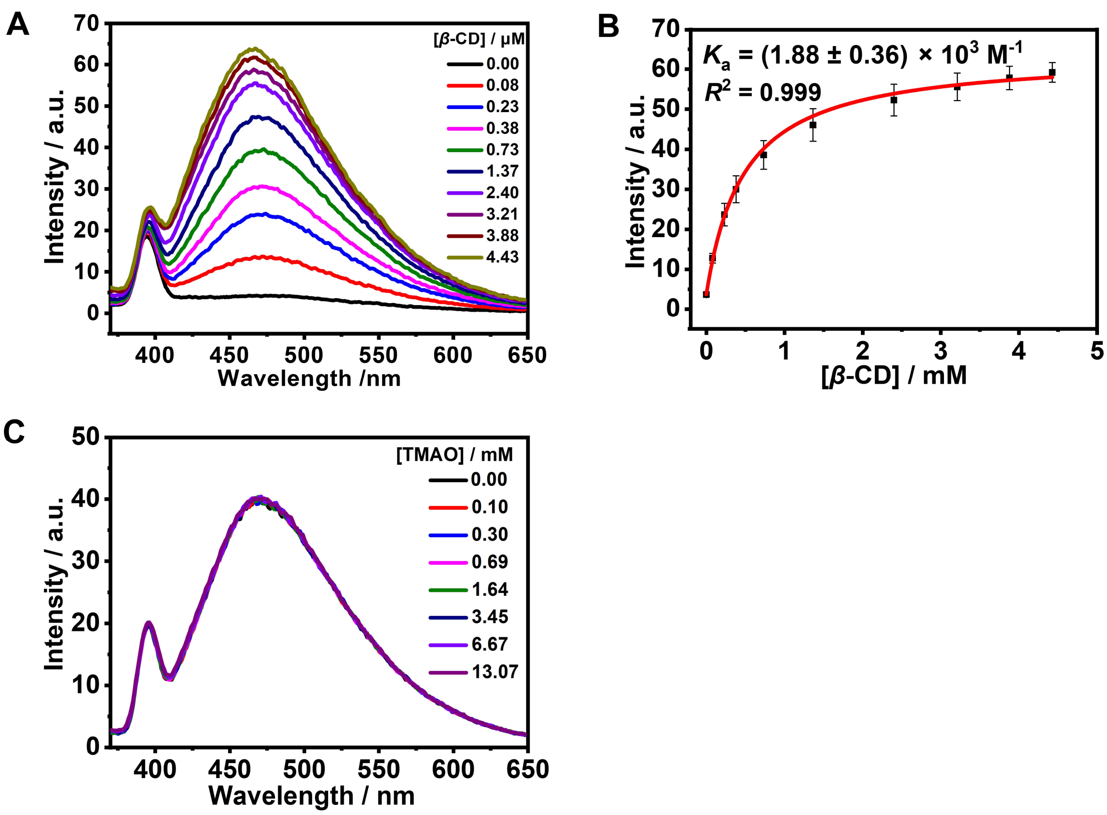

Figure S2. Direct fluorescence titration of 2,6-TNS (10.00 μM) with β-CD (up to 4.43 μM) at λ_{ex} = 350 nm (A), titration curve (λ_{em} = 483 nm) acquired by a 1:1 binding model (B), and competitive titration in the β-CD•2,6-TNS (1.00 mM/10.00 μM) reporter pair with TMAO (up to 13.07 mM) at λ_{em} = 483 nm (C). All experiments were in HEPES buffer (10 mM, pH 7.4) at 25 °C. Error bars smaller than 0.005 were not shown.

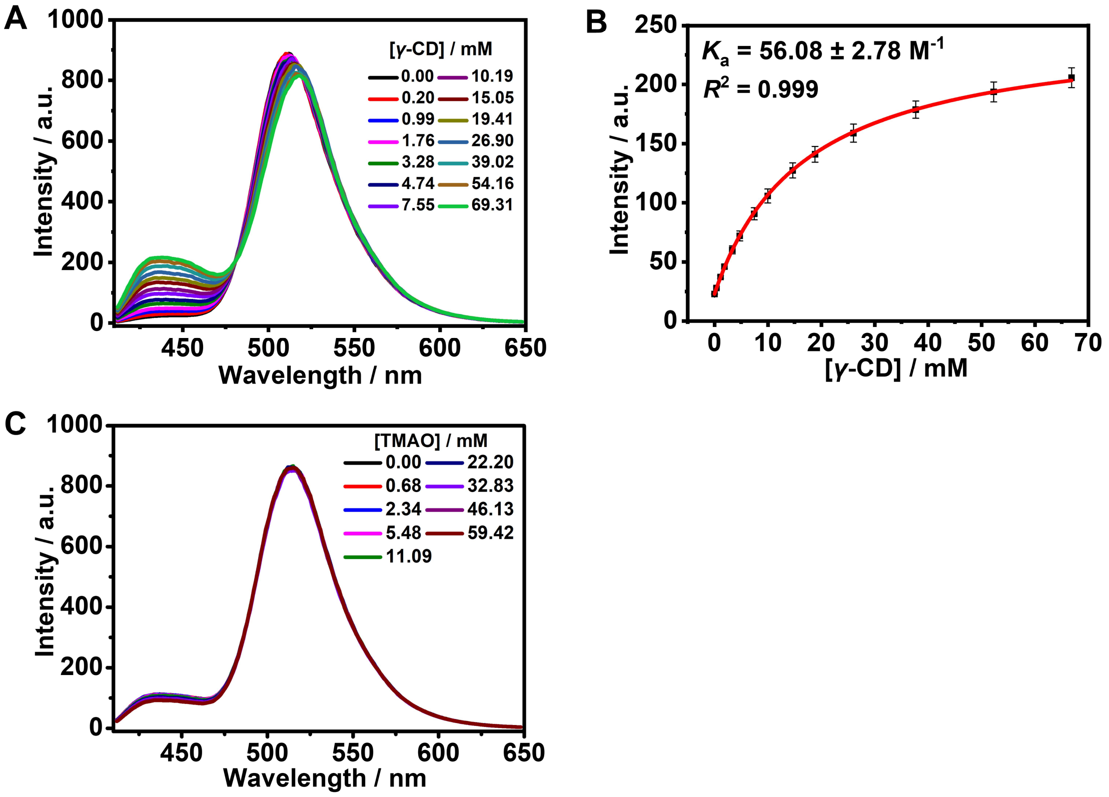

Figure S3. Direct fluorescence titration of HPTS (10.00 μM) with γ-CD (up to 69.31 mM) at λ_{ex} = 405 nm (A), titration curve (λ_{em} = 435 nm) acquired by a 1:1 binding model (B), and competitive titration in the γ-CD•HPTS (10.00 mM/10.00 μM) reporter pair with TMAO (up to 59.42 mM) at λ_{em} = 435 nm (C). All experiments were in HEPES buffer (10 mM, pH 7.4) at 25 °C. Error bars smaller than 0.005 were not shown.

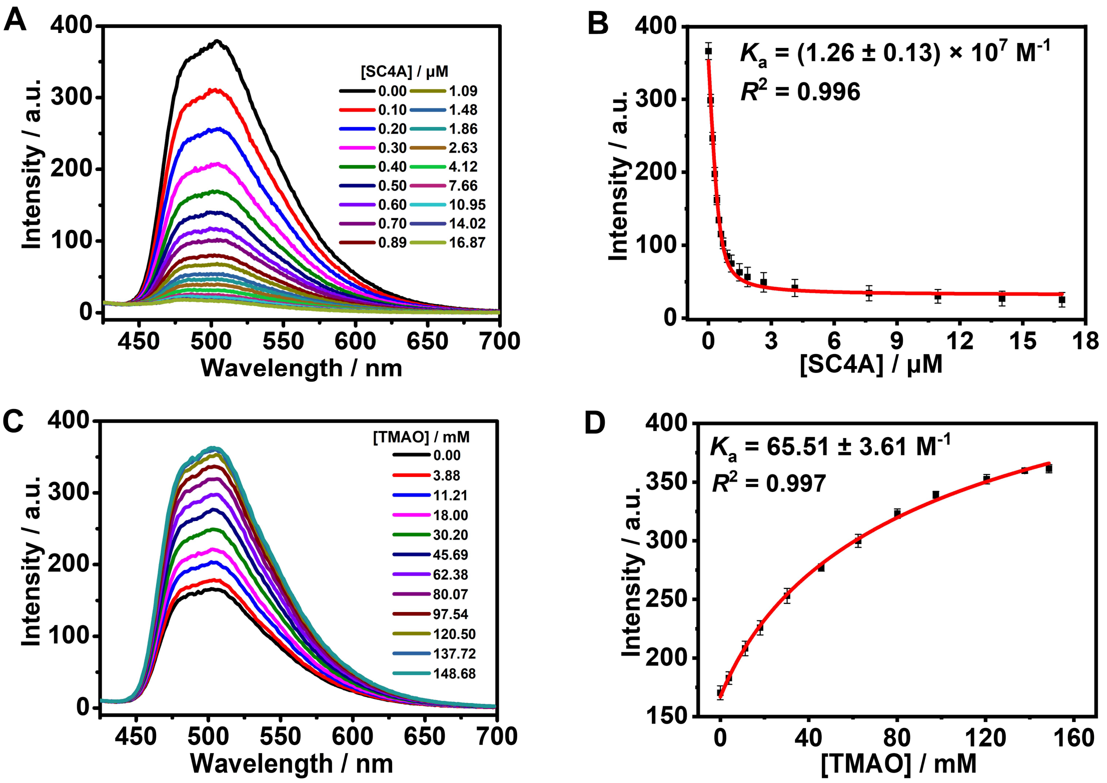

Figure S4. Direct fluorescence titration of LCG (0.50 μM) with SC4A (up to 16.87 μM) at λ_{ex} = 368 nm (A), and titration curve (λ_{em} = 505 nm) acquired by a 1:1 binding model (B). The competitive titration in the SC4A•LCG (0.50/0.50 μM) reporter pair with TMAO (up to 148.68 mM) (C), and titration curve (λ_{em} = 505 nm) acquired by a 1:1 competitive binding model (D). All experiments were in HEPES buffer (10 mM, pH 7.4) at 25 °C. Error bars smaller than 0.005 were not shown.

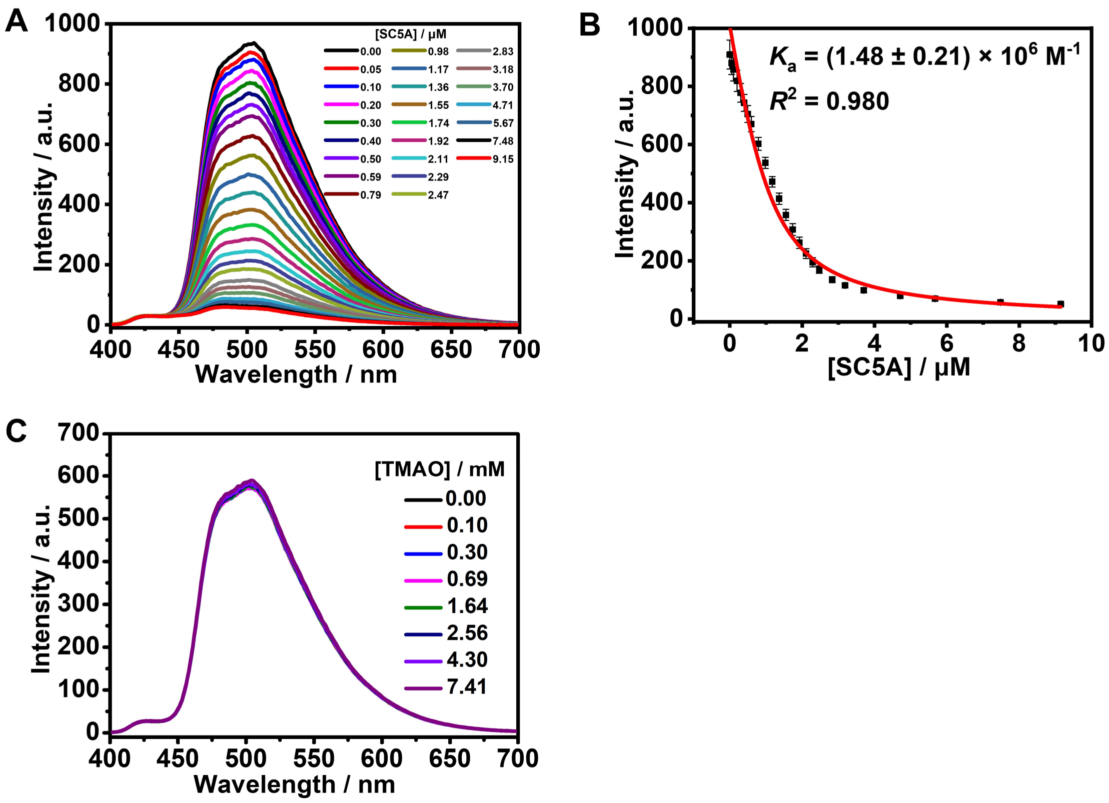

Figure S5. Direct fluorescence titration of LCG (1.00 μM) with SC5A (up to 9.15 μM) at λ_{ex} = 368 nm (A), titration curve (λ_{em} = 505 nm) acquired by a 1:1 binding model (B), and competitive titration in the SC5A•LCG (1.00/1.00 μM) reporter pair with TMAO (up to 7.41 mM) at λ_{em} = 505 nm (C). All experiments were in HEPES buffer (10 mM, pH 7.4) at 25 °C. Error bars smaller than 0.005 were not shown.

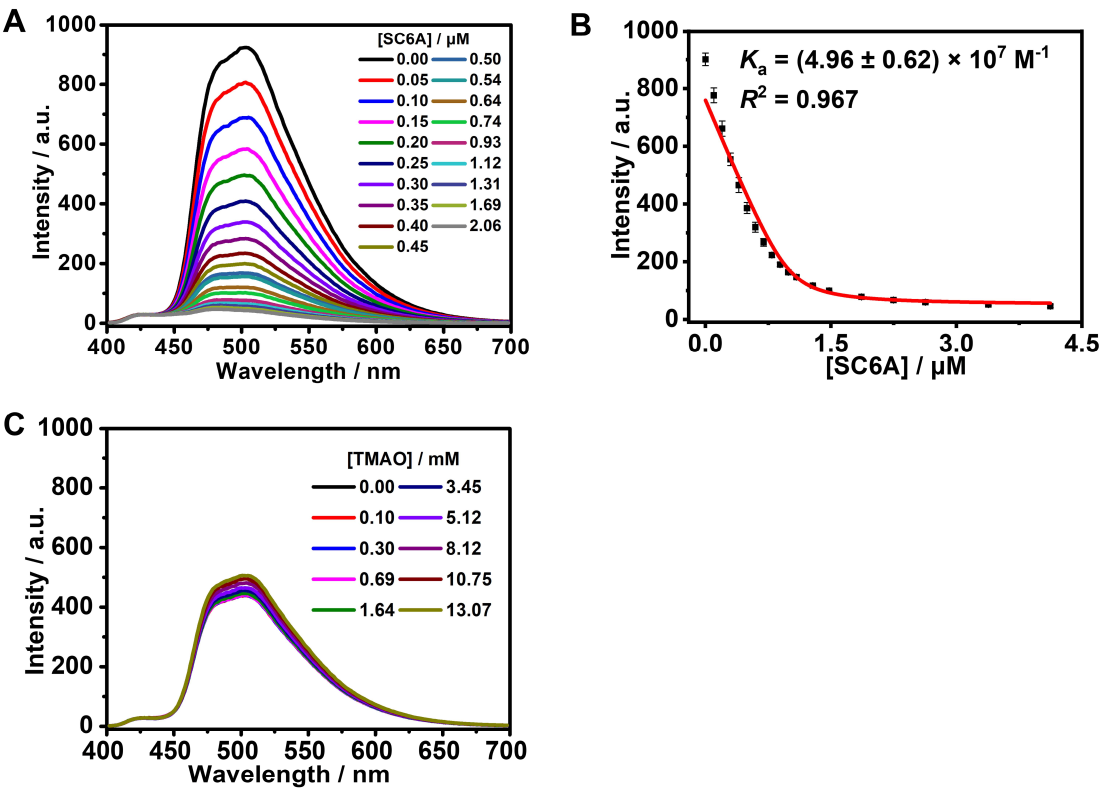

Figure S6. Direct fluorescence titration of LCG (1.00 μM) with SC6A (up to 2.06 μM) at λ_{ex} = 368 nm (A), titration curve (λ_{em} = 505 nm) acquired by a 1:2 binding model (B), and competitive titration in the SC6A•LCG (0.25/1.00 μM) reporter pair with TMAO (up to 13.07 mM) at λ_{em} = 505 nm (C). All experiments were in HEPES buffer (10 mM, pH 7.4) at 25 °C. Error bars smaller than 0.005 were not shown.

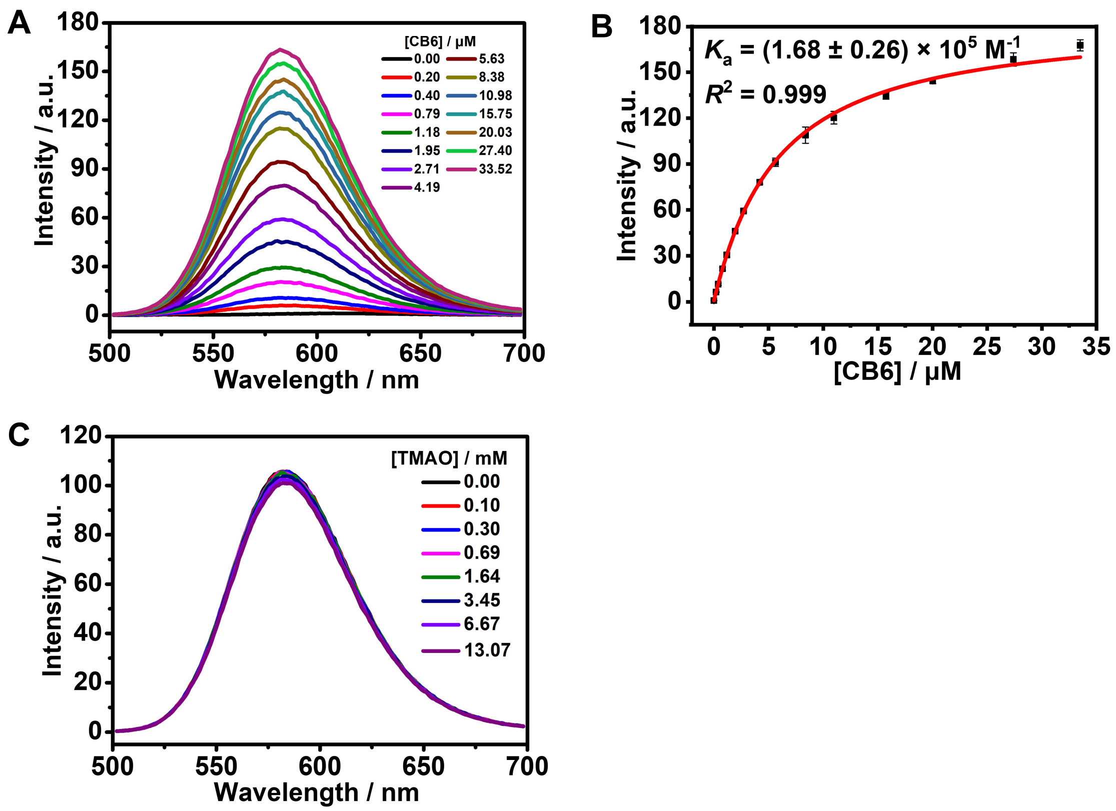

Figure S7. Direct fluorescence titration of DSMI (1.00 μM) with CB6 (up to 33.52 μM) at λ_{ex} = 450 nm (A), titration curve (λ_{em} = 582 nm) acquired by a 1:1 binding model (B), and competitive titration in the CB6•DSMI (8.00/1.00 μM) reporter pair with TMAO (up to 13.07 mM) at λ_{em} = 582 nm (C). All experiments were in HEPES buffer (10 mM, pH 7.4) at 25 °C. Error bars smaller than 0.005 were not shown.

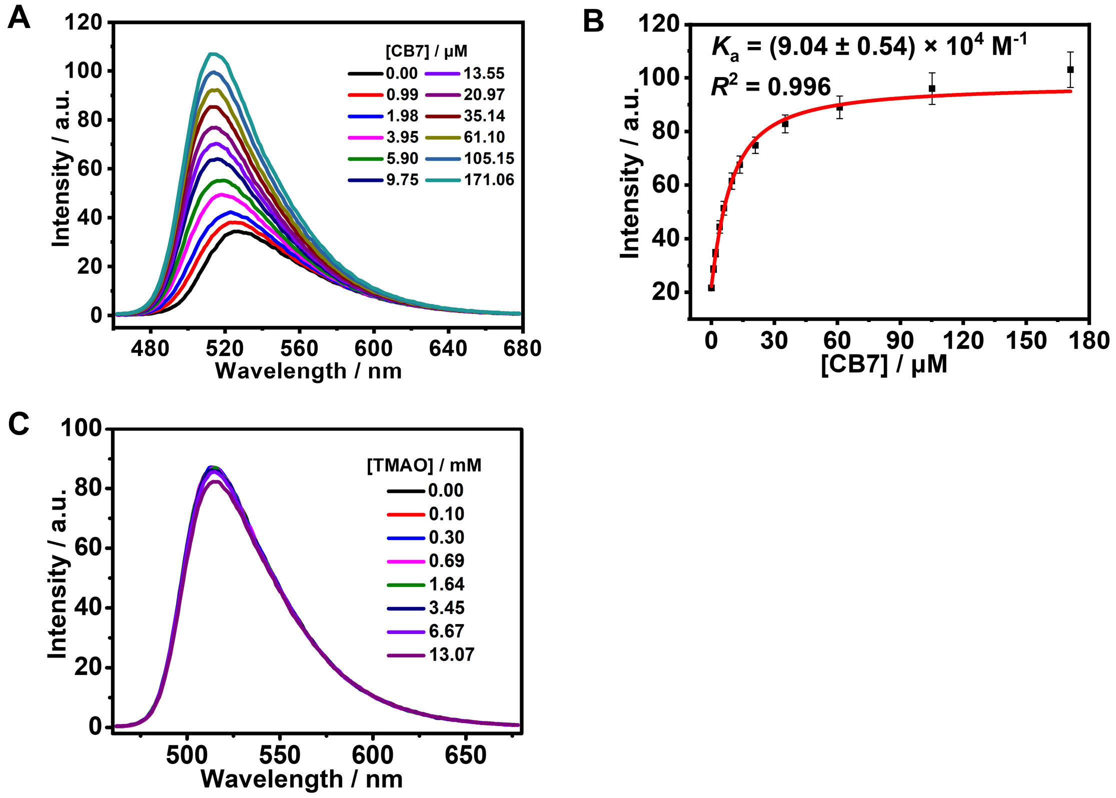

Figure S8. Direct fluorescence titration of AO (0.50 μM) with CB7 (up to 171.06 μM) at λ_{ex} = 450 nm (A), titration curve (λ_{em} = 510 nm) acquired by a 1:1 binding model (B), and competitive titration in the CB7•AO (15.00/0.50 μM) reporter pair with TMAO (up to 13.07 mM) at λ_{em} = 510 nm (C). All experiments were in HEPES buffer (10 mM, pH 7.4) at 25 °C. Error bars smaller than 0.005 were not shown.

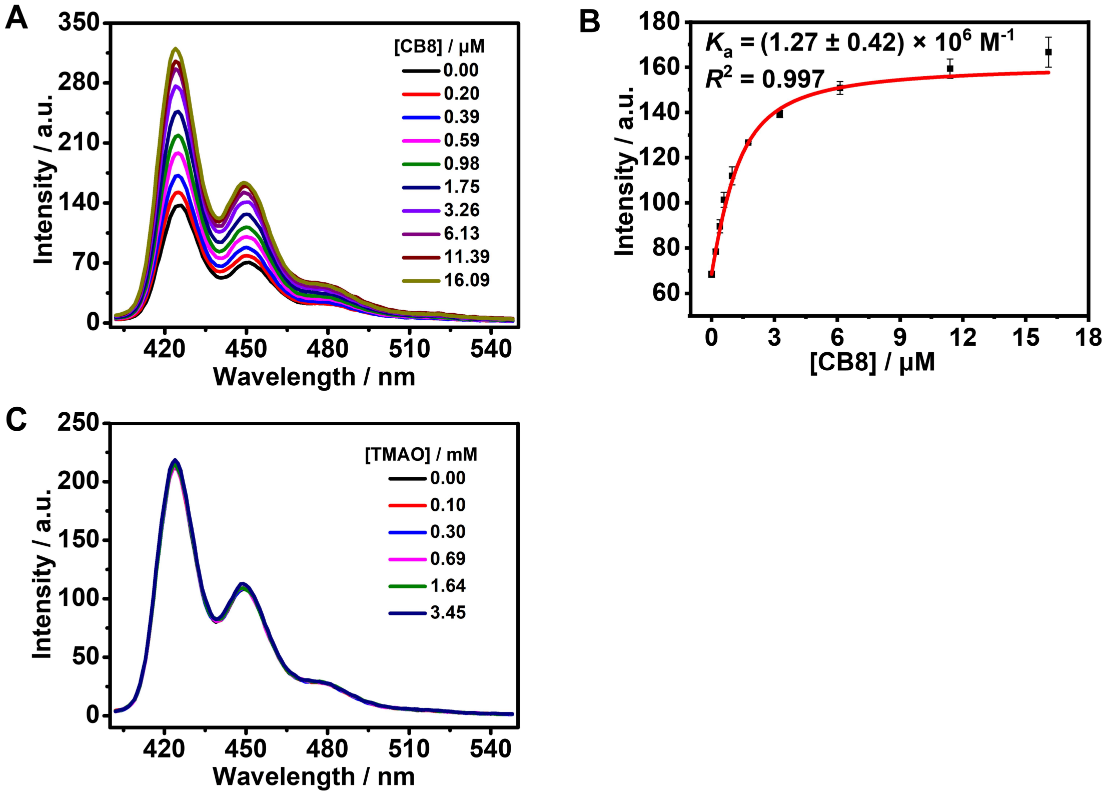

Figure S9. Direct fluorescence titration of Me_2DAP (1.00 μM) with CB8 (up to 16.09 μM) at λ_{ex} = 335 nm (A), titration curve (λ_{em} = 449 nm) acquired by a 1:1 binding model (B), and competitive titration in the CB8•Me_2DAP (2.00/1.00 μM) reporter pair with TMAO (up to 3.45 mM) at λ_{em} = 449 nm (C). All experiments were in HEPES buffer (10 mM, pH 7.4) at 25 °C. Error bars smaller than 0.005 were not shown.

2. Direct fluorescence titration of Fl with TMAO

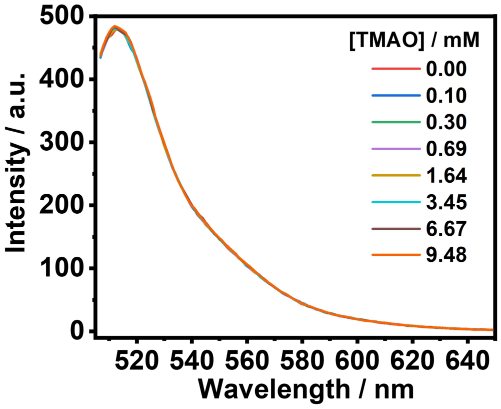

Figure S10. Direct fluorescence titration of Fl (1.00 μM) with TMAO (up to 9.48 mM) at λ_{em} = 513 nm (λ_{ex} = 500 nm) in 10 mM HEPES buffer solution (pH 7.4) at 25 °C.

3. 2D ROESY spectrum of GC5A•TMAO

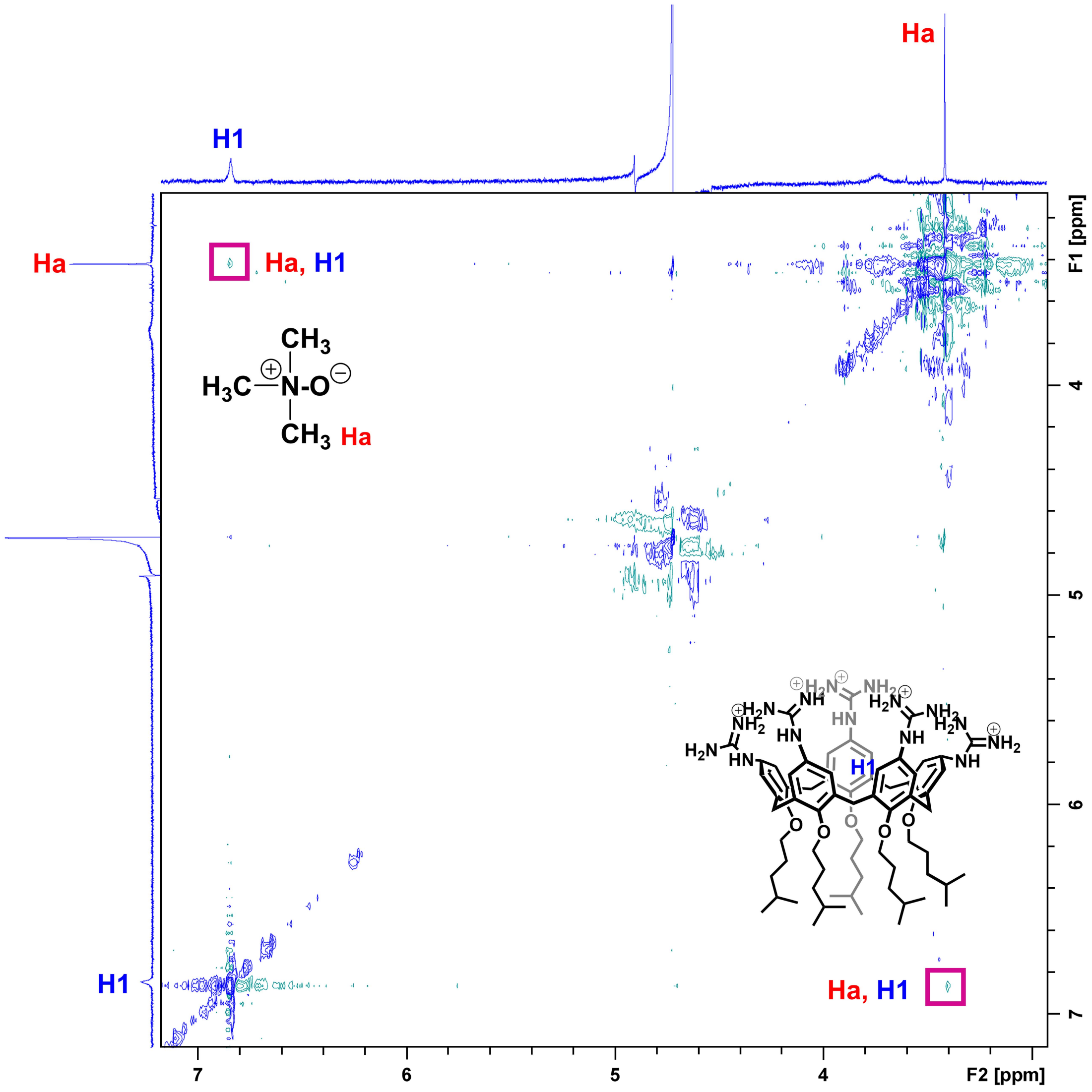

Figure S11. Section of the 2D ROESY spectrum (400 MHz, D_2O, 298 K) of GC5A•TMAO.

4. The limit of detection (LOD) for TMAO in HEPES buffer solution

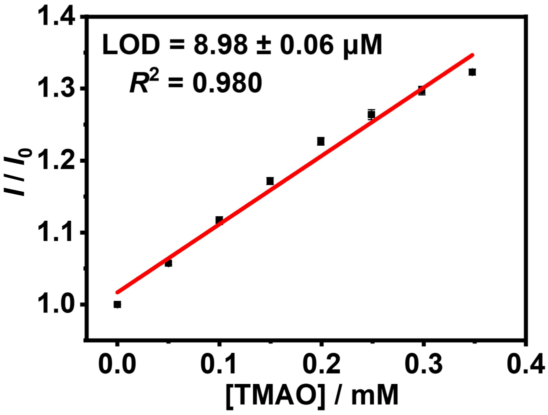

Figure S12. Plot of I/I_0 against TMAO concentration in 10 mM HEPES buffer solution (pH 7.4) at 25 °C, where I and I_0 were assigned as the fluorescence intensities of the GC5A•Fl (0.80/1.00 μM) reporter pair in the presence and absence of TMAO (0 – 0.35 mM), respectively. Error bars smaller than 0.005 were not shown.
